# Biological and Pro-Angiogenic Properties of Genetically Modified Human Primary Myoblasts Overexpressing Placental Growth Factor in In Vitro and In Vivo Studies

**DOI:** 10.1007/s00005-017-0486-2

**Published:** 2017-09-26

**Authors:** Agnieszka Zimna, Bartosz Wiernicki, Tomasz Kolanowski, Natalia Rozwadowska, Agnieszka Malcher, Wojciech Labedz, Tomasz Trzeciak, Katarzyna Chojnacka, Katarzyna Bednarek-Rajewska, Przemyslaw Majewski, Maciej Kurpisz

**Affiliations:** 1 Institute of Human Genetics, Polish Academy of Sciences, Strzeszynska 32, 60-479 Poznan, Poland; 2 Department of Orthopaedics and Traumatology, W. Dega University Hospital, Poznan University of Medical Sciences, Poznan, Poland; 3 Department of Clinical Pathomorphology, H. Swiecicki University Hospital, Poznan University of Medical Sciences, Poznan, Poland

**Keywords:** Regenerative medicine, Stem cell therapy, Myocardial infarction, Human skeletal myoblasts, Placental growth factor

## Abstract

Cardiovascular diseases are a growing problem in developing countries; therefore, there is an ongoing intensive search for new approaches to treat these disorders. Currently, cellular therapies are focused on healing the damaged heart by implanting stem cells modified with pro-angiogenic factors. This approach ensures that the introduced cells are capable of fulfilling the complex requirements of the environment, including the replacement of the post-infarction scar with cells that are able to contract and promote the formation of new blood vessels that can supply the ischaemic region with nutrients and oxygen. This study focused on the genetic modification of human skeletal muscle cells (SkMCs). We chose myoblast cells due to their close biological resemblance to cardiomyocytes and the placental growth factor (*PlGF*) gene due to its pro-angiogenic potential. In our in vitro studies, we transfected SkMCs with the *PlGF* gene using electroporation, which has previously been proven to be efficient and generate robust overexpression of the *PlGF* gene and elevate PlGF protein secretion. Moreover, the functionality of the secreted pro-angiogenic proteins was confirmed using an in vitro capillary development assay. We have also examined the influence of *PlGF* overexpression on *VEGF*-*A* and *VEGF*-*B*, which are well-known factors described in the literature as the most potent activators of blood vessel formation. We were able to confirm the overexpression of *VEGF*-*A* in myoblasts transfected with the *PlGF* gene. The results obtained in this study were further verified in an animal model. These data were able to confirm the potential therapeutic effects of the applied treatments.

**Electronic supplementary material:**

The online version of this article (doi:10.1007/s00005-017-0486-2) contains supplementary material, which is available to authorized users.

## Introduction

Cardiovascular disease is a widespread and growing problem. Disorders that fall under this category include cardiomyopathies, and these are the most frequent diseases in western countries (World Health Organization 2012, http://www.who.int/topics/cardiovascular_diseases/en/index.html; Roger et al. 2012). During myocardial infarction, the reduction in blood supply leads to the atrophy of cardiomyocytes. Subsequently, the ischaemic region becomes occupied with fibroblasts and endothelial cells, resulting in the formation of a post-infarction scar that has a limited ability to contract. This process may be a substantial contributor to left ventricle remodelling and the subsequent deterioration towards heart failure (Haider et al. 2009). Cellular therapies are emerging as a novel potential therapeutic approach for many disorders. A search for more effective therapies has led to the application of stem cells as a pro-regenerative tool to heal damaged heart tissues. The restoration of the myocardium is feasible by the use of stem cells with similar properties to those of cardiomyocytes; for example, myoblasts give rise to myogenic cell precursors of other origin. Human skeletal muscle stem cell (SkMC)-derived myoblasts are able to contract, and these cells are exceptionally resistant to ischaemia and hypoxia. The close resemblance of SkMCs to cardiomyocytes has been established; therefore, myoblasts have been frequently employed in the treatment of heart failure (Gavira et al. 2008; Menasché 2007, 2008a, b).

Therapies using “native” stem cells are often incapable of inducing sufficient angiogenesis; however, these cells can still support the failing heart. To improve the effect of engrafted cells, we propose a combined stem cell and gene therapy. There are several pro-angiogenic factors that stimulate capillary formation (Kawasuji 2002), with vascular endothelial growth factor (VEGF) having the greatest potential as a direct effector in neoangiogenesis (Becker et al. 2006; Rissanen and Ylä-Herttuala 2007). In this study, we solely focused on a placental growth factor (PlGF) isoform 1 because of its secretory ability. Introduction of this gene of interest into myoblast cells results in its overexpression, and in theory the secretion of pro-angiogenic proteins in the local environment should lead to an increase in blood vessel development (Maglione et al. 1991). It has been shown that PlGF participates in angiogenesis through four different mechanisms: (1) by indirectly enhancing the activity of VEGF-A, which leads to the stimulation of endothelial cell growth and migration; (2) by stimulating smooth muscle cells and thereby promoting vessel maturation and stabilization; (3) by recruiting inflammatory cells that play a crucial role in capillary growth and (4) by mobilizing progenitor cells from the bone marrow (Autiero et al. 2003). Forced overexpression of *PlGF* in myoblast cells may support the in vivo therapeutic effect, as this method was demonstrated in previous studies in rats with the in vivo delivery of myoblasts overexpressing the *PlGF* gene to the infarcted heart. The resulting capillary growth in this model was significantly enhanced (Gmeiner et al. 2011). Overproduction of pro-angiogenic proteins may contribute to angiogenesis by the individual interaction of these proteins with other factors, which may lead to augmentation of this process (Cao et al. 1996; Gigante et al. 2006; Roncal et al. 2008).

The principal aim of this study was to obtain a population of human myoblasts with exogenous *PlGF* gene overexpression. Our first goal was to examine the biological properties of these genetically modified myoblasts in vitro. We determined the effect of *PlGF* overexpression on cell proliferation, as well as its angiogenic potential and influence on myogenic gene expression and cell viability under oxidative stress conditions. The second goal of this study was to evaluate the effects of *PlGF*-transfected myoblasts on post-infarcted hearts using a mouse model.

## Materials and Methods

### Cells Studied

Human myoblasts were obtained from tissue fragments derived from a skeletal muscle biopsy. For this purpose, approval from the local bioethical committee was required. Isolation of muscle stem cells was performed according to a previously described pre-plate technique with modifications (Rozwadowska et al. 2002). The cells were analysed by flow cytometry to establish the purity of the obtained population. Myoblasts were identified using an antibody against the myogenic marker CD56. Further verification was performed via staining for desmin, a protein characteristically expressed in myogenic cells. Cells were cultured in standard Dulbecco’s modified Eagle’s medium with 4.5 g/l of glucose supplemented with 20% foetal bovine serum (FBS), 1% antibiotics, 1% ultraglutamine (Lonza Group, Basel, Switzerland) and other routine supplements. Additionally, bFGF (Sigma-Aldrich, St. Louis, USA) was added to the medium as previously described (Rozwadowska et al. 2002).

Human umbilical vein endothelial cells (HUVECs) were required to evaluate the pro-angiogenic properties of PlGF. Cells were maintained in endothelial basal medium supplemented with 10% foetal calf serum, hydrocortisone (1 µg/ml), bovine brain extract (3 µg/ml), gentamicin (50 µg/ml), amphotericin B (50 µg/ml) and epidermal growth factor (10 µg/ml).

Cells were maintained under standard in vitro conditions with 95% humidity and 5% CO_2_ at 37 °C. The medium was changed every 2 days, and the cells were passaged using 0.25% trypsin with EDTA (Sigma-Aldrich, St. Louis, USA). The procedure was performed at approximately 70–80% confluency, which was estimated by microscopic observations.

### Plasmids

The *PlGF* coding sequence was amplified from the cDNA template obtained from HUVECs. PCR was performed using primers designed for the first isoform of the *PlGF* coding sequence derived from the BLAST database (all primers used in this study are listed in Table 1). Then, the PCR product was cloned into the pSC-A (Strataclone kit-Promega, Madison, USA) vector. To achieve *PlGF* expression in mammalian cells, the insert from the primary plasmid was subcloned into the pCi-neo (Promega, Madison, USA) vector using the restriction enzymes *Xho*I and *Xba*I.

**Table 1:** Primer sequences used in real-time PCR

Gene name	Primer name	Primer sequence	Product size (bp)
*hACTβ*	ACTβ_f	5′-GCTGTATGAGACATCCCCCTA-3′	
	ACTβ_r	5′-ATCTTGATCTTCATTGTGCTG-3′	192
*hPlGF*	PlGF_f	5′-GGCTCGTCAGAGGTGGAAGT-3′	
	PlGF_r	5′-GCAGCAGGGAGACACAGGAT-3′	145
*hPlGF_orf*	PlGF _f	5′-CTCGAGCTGAGAAGATGCCGGTCATGAGGCTGTT-3′	
	PlGF_r	5′-TCTAGAAGCCGGGTGCGGGGTCTCTCTCCTCCAAG-3′	514
*mPlgf*	mPlgf_f	5′-GTGTCCTTCTGAGTCGCTGT-3′	
	mPlgf_r	5′-CCTTTCTGCCTTTGTCGTCT-3′	197
*hMYOG*	MYOG_f	5′-GCTGTATGAGACATCCCCCTA-3′	
	MYOG_r	5′-CGACTTCCTCTTACACACCTTAC-3′	226
*hMYF5*	MYF5_f	5′-TGCAGGAGTTGCTGAGAGAGCA-3′	
	MYF5_r	5′-CAGGACTGTTACATTCGG-3′	120
*hMYF6*	MYF6_f	5′-CTTCAGCTACAGACCCAAACA-3′	
	MYF6_r	5′-CCTGGAATGATCGGAAACAC-3′	94
*hMYOD*	MYOD_f	5′-ACGGCATGATGGACTACAG-3′	
	MYOD_r	5′-CGACTCAGAAGGCACGTC-3′	212
*hMEF2*	MEF2_f	5′-AGATACCCACAACACACGCG-3′	
	MEF2_r	5′-ATCCTTCAGAAAGTCGCATGC-3′	193
*hVEGF*-*A*	VEGF-A_f	5′-AAGGAGGAGGGCAGAATCAT-3′	
	VEGF-A_r	5′-CACACAGGATGGCTTGAAGA-3′	143
*hVEGF*-*B*	VEGF-B_f	5′-AGTGGGGGAACAAAGAGGAG-3′	
	VEGF-B_r	5′-TCAGGGAGACAAGGGATGG-3′	123
*mVegf*-*a*	mVegf-a_f	5′-TCCTGTGTGCCGCTGATG-3′	
	mVegf-a_r	5′-GCTGGCTTTGGTGAGGTTTG-3′	121
*mVegf*-*b*	mVegf-b_f	5′-GTCGCCTGCTGCTTGTTG-3′	
	mVegf-b_r	5′-ACTTTCTTCTGGTGGCTGGG-3′	88
*mVegf*-*c*	mVegf-c_f	5′-AGATGTGGGGAAGGAGTTTG-3′	
	mVegf-c_r	5′-ACTGATTGTGACTGGTTTGGG-3′	193
*mVegf*-*d*	mVegf-d_f	5′-CTGGGGAAGACAACCAACAC-3′	
	mVegf-d_r	5′-CAGGCACTAACTCGGGCA-3′	172
*mFlt*-*1*	mFlt-1_f	5′-GCTTTCACCGAACTCCACCT-3′	
	mFlt-1_r	5′-AGTCCCGCCTCCTTGCTTT-3′	162
*mKdr*	mKdr_f	5′-TCTGGACTCTCCCTGCCTAC-3′	
	mKdr_r	5′-CGGCTCTTTCGCTTACTGTTC-3′	128

*MYF5* myogenic factor 5, *MYOD* myogenic differentiation 1, *MYOG* myogenin, *MRF4* (*MYF6*) myogenic factor 6, *MEF2* myocyte enhancer factor 2, *PlGF* placental growth factor, *ACTB* beta actin, *VEGF* vascular endothelial growth factor (isoform A and B; *h* human), *mVegf a, b, c, d* mouse vascular endothelial growth factor isoforms, *mPlgf* mouse placental growth factor, *mKdr* mouse kinase insert domain receptor, *mFlt-1* mouse fms-related tyrosine kinase I receptor

To determine the efficiency of the transfection process, we used the pEGFP-C1 (Clontech, Mountain View, USA) plasmid, which contains the coding sequence of green fluorescent protein (GFP).

### *PlGF* Gene Transfer

The *PlGF* gene was introduced into human myoblasts by electroporation. Previous optimization of this method allowed us to achieve a high efficacy of gene transduction. The electroporation conditions were as follows: one pulse for 15 ms, wave tension of 160 V and cuvettes with a 2-mm gap. To successfully transduce the expected number of cells, we used 3 × 10^6^ myoblasts resuspended in a final volume of 200 µl of F10 medium; the plasmid concentration was established according to plasmid length.

The medium was changed 24 h after electroporation. The transfection efficacy was evaluated 48 h after electroporation. For this purpose, we used myoblasts transfected with the pEGFP-C1 plasmid. Two assessments were performed: first, we observed GFP fluorescence in the transfected cells under an inverted microscope (Zeiss Axiovert 200 microscope), and second we performed cell staining with propidium iodide to identify necrotic cells. Using flow cytometry (Beckman Coulter, Fullerton, USA), we determined the number of GFP-positive and PI-positive cells to establish the transfection efficacy and cell viability. To obtain the highest yield, a separate transfection was performed for each planned experiment. GFP transfection was conducted in parallel to the electroporation of *PlGF* to monitor its expression.

### PlGF Immunoassay

To determine the concentration of the secreted PlGF protein, we performed a quantitative ELISA (R&D System, Abingdon, UK). The supernatants from cultured cells (transfected and “native”) were harvested 48 h after electroporation. The experiments were performed according to the manufacturer’s instructions.

### Expression of VEGF Isoforms in Transfected Myoblasts

To assess the expression of the *VEGF*-*A* and *VEGF*-*B* genes, real-time PCR analysis was performed. First, we obtained two plasmid constructs containing these isoforms to construct a standard curve for each gene to verify the data obtained by real-time PCR with respect to the relative expression of the studied gene. Both isoforms were derived by PCR with primers specific for the sequences of *VEGF*-*A* and *VEGF*-*B*. As a template for PCR, we decided to use cDNA derived from HUVECs. After PCR, the products were separated by gel electrophoresis and purified using appropriate columns (GeneJET Extraction Kit, Thermo Scientifics, Lafayette, USA). These sequences were then cloned into the pSC-A vector using the Strata Clone Kit (Promega, Madison, USA) and subsequently isolated from overnight bacterial culture.

### Cell Proliferation

The proliferation of *PlGF*-transfected cells was assessed using the Cell Proliferation Kit I (MTT) (Roche, Mannheim, Germany). The test was based on the ability of metabolically active cells to convert tetrazolium salt to purple formazan crystals. First, the genetically modified and “native” cells were seeded in 96-well flat-bottom adherent plates at a final density of 2.5 × 10^3^ cells per well and cultured for 72 h. Then, the assay was performed in triplicate. For detection, an ELISA plate reader (BioTek Instruments Inc, USA) was used.

### Detection of Cell Apoptosis

Using an Annexin V-FITC Kit (Beckman Coulter, Fullerton, USA), we detected the percentage of apoptotic cells in the examined myoblast populations. The assessment is based on the ability of Annexin V to bind phosphatidylserine. These phospholipids become exposed to the cell surface during early stages of apoptosis. Annexin V is linked to FITC (fluorescein) and labels apoptotic cells. The analysis was performed using the Cell Lab Quanta MPL flow cytometer. To evaluate the influence of oxidative stress cells were assessed in parallel under oxidative stress conditions. Oxidative stress was induced by the addition of 500 µM H_2_O_2_ to the culture medium. Myoblasts were incubated with hydrogen peroxide for 24 h after which the apoptosis levels were examined.

### Pro-Angiogenic Assays

To determine the pro-angiogenic properties of the PlGF protein, we performed two assays based on the proliferation of HUVECs. For both assays, the supernatants from the genetically modified and “native” myoblast cultures were harvested 48 h after electroporation. First, the sprouting test was used to measure the total length of the capillaries sprouting from the HUVEC aggregates. To obtain a spheroid culture of HUVECs, the cells were seeded in 96-well U-bottom plates (Greiner, Frickenhausen, Germany) on a scaffold made from methylcellulose. After overnight incubation, the aggregates were harvested and centrifuged. Next, the spheroids were gently mixed with the solution containing 30% FBS in methylcellulose. Meanwhile, collagen was neutralized using 0.2 M NaOH. Then, the collagen was added to the HUVEC aggregates, and the resultant mixture was seeded in 24-well plates and incubated for 30 min at 37 °C. Supernatants from transfected myoblasts were then harvested and transferred to the spheroid cultures. Assessment of capillary length was performed 48 h after the transfer of the media. Each spheroid was photographed, and the length of every sprouting capillary was measured and summarized for each spheroid. After measuring each sprouting capillary for every spheroid culture in the examined variants, the mean value was calculated. This procedure was repeated for every variant in the study. Second, the pseudotube formation assay in Matrigel was performed. HUVECs were cultured (for no more than four passages) at approximately 80–90% confluency using Medium 200 supplemented with Large Vessel Endothelial Supplement (LVES) (Gibco, Carlsbad, USA). Then, the cells were trypsinized, counted and seeded at a density of approximately 25,000 cells per cm^2^ on 24-well plates coated with Geltrex Matrix (Gibco, Carlsbad, USA). The supernatants collected from the different myoblast cultures were transferred to the prepared HUVECs to evaluate the functional properties of the secreted proteins. As a negative control, DMEM and fresh myoblast media were used; as a positive control, Medium 200 supplemented with LVES was used. The HUVECs were further incubated for 14–18 h with the supernatants harvested from myoblasts. Afterwards, the newly formed capillaries were stained with 2 μg/ml calcein-AM (Invitrogen, Carlsbad, USA).

### Myogenic Gene Expression Profiling

To assess the myogenic gene expression, real-time PCR was performed. To obtain the cDNA sample, we isolated total RNA using TRIzol reagent (Invitrogen, Carlsbad, USA) from genetically modified and “native” cells. A portion of the mRNA sample was purified using Oligo(dT)-Dynabeads (Invitrogen, Carlsbad, USA). Finally, reverse transcription of the mRNA samples was performed to generate cDNA samples. Quantitative assessment of myogenic gene expression was performed on an iCycler (BioRad, Hercules, USA). Primers were designed for the following myogenic genes: *MYOD, MYF5, MYOGENIN, MYF6* and *MEF2*, and purchased from Oligo, Warsaw, Poland (sequences for all of the primers used in the study are listed in Table 1). Gene expression was normalized using beta actin as a reference gene. The specificity of the real-time products was determined using melting curves and agarose gel electrophoresis.

### Animal Model

Animal experiments were performed on non-obese diabetic/severe combined immunodeficient (NOD-SCID) mice. This strain provides limited adaptive immunity and a low number of NK cells, which decreases the possibility of rejection of xenografts.

### Experimental Design

Experiments were performed with 15 post-infarction animals divided into three subgroups: transplanted with *PlGF*-transfected myoblasts (Mb-*PlGF*), *n* = 7; transplanted with wild-type myoblasts (Mb-Wt), *n* = 4; and injected with 0.9% NaCl, *n* = 4. Before ligation of the left coronary artery, cardiac parameters were assessed by echocardiography across all animal groups. At 17 days after the induction of heart infarction, the animals were examined by echocardiography once again to confirm the occurrence of myocardial infarction (MI). At 28 days after coronary artery ligation, either cells (Mb-*PlGF* and Mb-Wt) or 0.9% NaCl was injected into the infarction border zone (3 × 10 µl, 3 × 10^5^ cells/injection). After another 10 days, cardiac parameters were measured by echocardiography to observe the early effects of cell transplantation. Long-term effects of the transplant therapy were assessed 2 months after cell transplantation by echocardiography. Subsequently, the animals were euthanized and the hearts were collected.

### Induction of Heart Infarction, Cell Transplantation and echocardiography

All experiments involving animals were approved by the local ethical committee for animal research in Poznan. Female NOD-SCID mice (12-week-old) were purchased from Jackson Laboratory (Bar Harbor, USA) and used in experiments. There were no significant changes in weight among the animals (data not shown). Briefly, the animals were anaesthetized with 2% isoflurane. Myocardial infarction was induced following left coronary artery ligation. The procedure was preceded by opening the thorax through the fourth intercostal space and removing the pericardium. After induction of the infarct, the thorax and skin were stitched, and the mice were ventilated with oxygen until they began to respirate independently.

Transplantation was performed 28 days after the induction of myocardial infarction. Cells for transplantation were cultured and genetically modified as described above (sections “Cells Studied” and “PlGF Gene Transfer”) under standard in vitro conditions. Before surgical injection, the cells were harvested by trypsinization, centrifuged (1200 rpm/RT) and resuspended in DMEM without phenol red. Cells were transplanted during the surgical procedure as described above and injected into three spots around the infarction border zone (3 × 10 µl, 3 × 10^5^ cells/injection).

Assessment of cardiac parameters was performed using a GE Vivid 7 high-resolution ultrasound scanner equipped with an M12L linear transducer (GE Healthcare, Chicago, USA). Before examination, the animals were anaesthetized with a xylazine/ketamine solution administered via intraperitoneal injection. Echocardiography was conducted at four time points: before MI, 17 days after heart infarction, and 10 days and 2 months after cellular/0.9% NaCl interventions. We measured the left ventricular end-systolic and end-diastolic areas in the short axis (LVESAS and LVEDAS, respectively) to determine the changes in the ratio of the left ventricular area (SAX AC%) using the following formula:

$$ {\text{SAX AC}}\% = \left( {\frac{{{\text{LVEDAS}} - {\text{LVESAS}}}}{\text{LVEDAS}}} \right) \times 100\% . $$

Using this formula, we could determine how the cardiac infarction and cell therapy influenced the left ventricular region and its subsequent contractility. After myocardial infarction, the contractility of the left ventricle is reduced; following this phenomenon, there are small differences in the area of the left ventricle under the diastole and systole that may lead to heart failure. After stem cell therapy, the contractility of the left ventricle should be improved, and the area under the diastole and systole may thus vary.

### Heart Tissue Collection and Histological and Gene Expression Analysis

The mice were euthanized via cervical dislocation at specific time points. One portion of the heart was prepared for immunohistochemical (IHC) staining by standard fixation and paraffin embedding, tissue sectioning and attachment of the sections on SuperFrost glass slides. To visualize the scar tissue, Masson’s trichrome staining was performed. The presence of cells transplanted into mouse infarcted heart was verified by IHC using an antibody against human mitochondria that does not cross-react with rat or mouse tissues (EMD Millipore, Billerica, USA). Slides were prepared for staining by 10 min of incubation in each of the reagents: xylene, 100% ethanol, 96% ethanol and 70% ethanol. Epitope retrieval was performed by heating the slides for 30 min at 95 °C in EDTA buffer containing 0.05% Tween 20, pH 8.0. For IHC staining, slides with exposed epitopes were incubated overnight with primary antibody. Subsequently, secondary antibody with Alexa Fluor 488 was used, and the slides were analysed using a fluorescence microscope.

The remaining portion of the isolated hearts was washed three times with a 0.9% NaCl solution. Next, the left ventricles were isolated, photographed and stored in RNAlater (Thermo Fisher, Carlsbad, USA) solution at −80 °C until further use. The collected ventricles were homogenized, and mRNA was isolated to examine gene expression after stem cell therapy. After reverse transcription of the mRNA, real-time PCR was performed, and the expression of the following genes was assessed: *Vegf*-*a, Vegf*-*b, Vegf*-*c, Vegf*-*d, Plgf* and the receptors *Kdr* and *Flt*-*1*.

### Statistical Analysis

All data were analysed with three tests: ANOVA with Tukey’s correction for unrelated variables, the Kruskal–Wallis test with Dunn’s correction for the comparison of multiple groups and the *χ* ^2^ test. The data are presented as the mean ± standard deviation.

## Results

### Modification of Human Myoblasts

Both markers CD56 and desmin were expressed at approximately 90% in the myoblast populations used in this study.

**Fig. 1:**
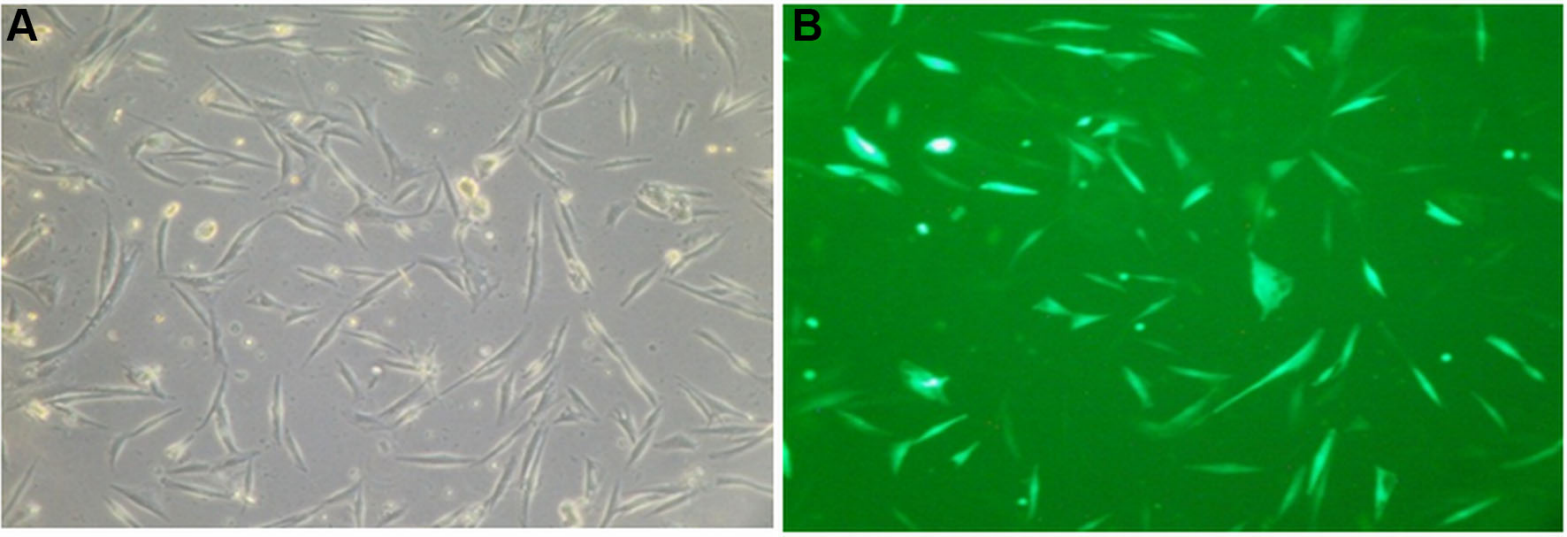
Cells transfected with plasmid containing GFP sequence were verified using flow cytometer with Cell Lab Quanta™ SC MPL (Beckman Coulter, Fullerton, USA) at 48 h after electroporation, showing approximately 80% efficacy. **a**, **b** the same population of myoblasts under light and fluorescence microscopes (×100 magnification), respectively

**Fig. 2:**
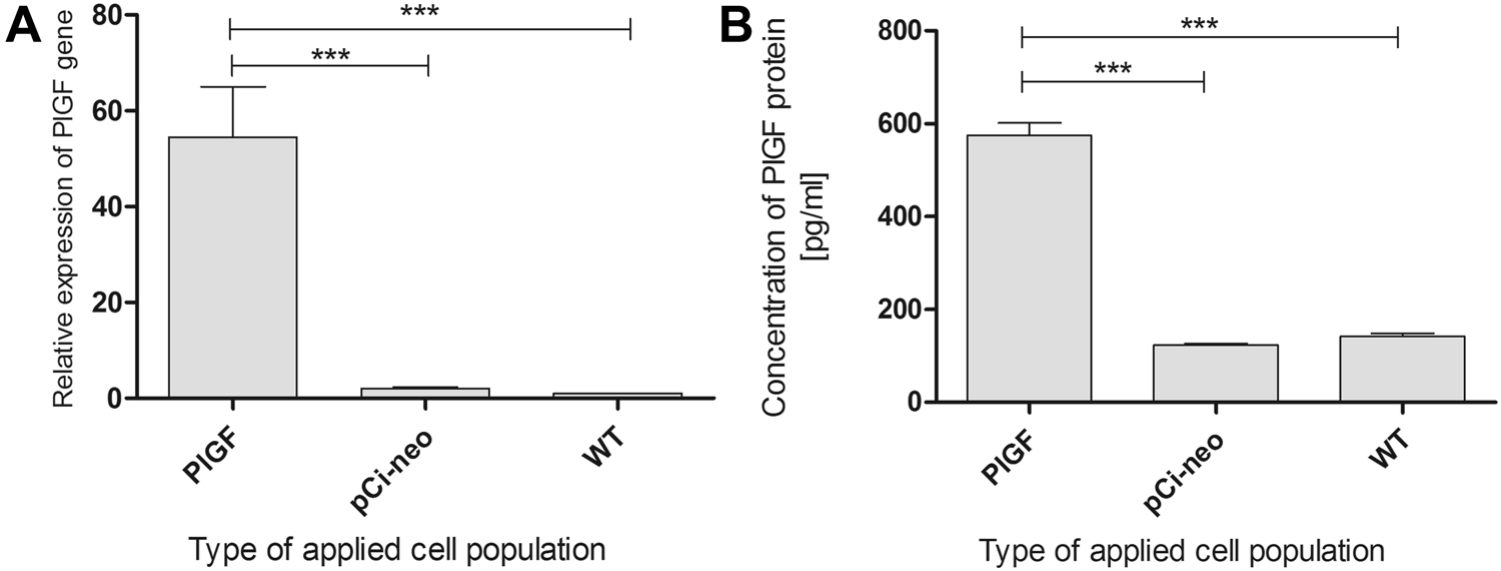
Relative expression of the examined gene was normalized according to the expression of the housekeeping β-actin gene. Data are presented as relative mRNA fold change in comparison with transcript level in wild-type cells. Experimental groups were as follows: *WT* non-transfected cells (wild-type cells), pCi-neo transfected control cells (myoblasts with plasmid without a coding sequence), *PlGF*-transfected cells containing the pCi-neo plasmid with coding sequence for the placental growth factor gene. **a** Expression of *PlGF* gene was significantly higher among the following populations: *PlGF*-transfected cells vs. pCi-neo transfected cells at *p* value, ***p < 0.001 and *PlGF*-transfected cells vs. WT cells at ***p < 0.001. Experiments were performed twice in triplicate. **b** Concentration of PlGF protein in culture supernatants harvested at 48 h after the transfection of human primary myoblasts, genetically modified cells (574.68 pg/ml ± 27.17), cells transfected with pCi-neo control vector (123.89 pg/ml ± 2.78) and native myoblasts—WT (142 pg/ml ± 6.94); *n* = 3 experiments. Statistically significant differences ***p < 0.001 between the following studied myoblast populations were found: *PlGF*-transfected cells vs. WT cells and *PlGF*-transfected cells vs. pCi-neo-transfected cells

Electroporation with a plasmid expressing GFP sequence was conducted in parallel with the electroporation of the pCi-neo-*PlGF* construct. Each transduction resulted in the uptake of the plasmids by approximately 80–90% of the cells (Fig. 1). It should also be mentioned that this method resulted in high cell mortality (approximately 40%), but it was the most efficient protocol that we have worked with thus far. Introducing the *PlGF* gene using electroporation triggered the overexpression of *PlGF* in genetically modified myoblasts, which was verified by real-time PCR. The elevated levels of *PlGF* transcripts (Fig. 2a) were translated into a high concentration of this pro-angiogenic protein (Fig. 2b). The expression of *PlGF* was 50-fold higher in the transduced cells than in the wild-type (WT) cells, and the amount of protein secreted into the medium reached almost 600 pg/ml, whereas “native” cells only secreted 142 pg/ml.

### Expression of the VEGF-A and VEGF-B

**Fig. 3:**
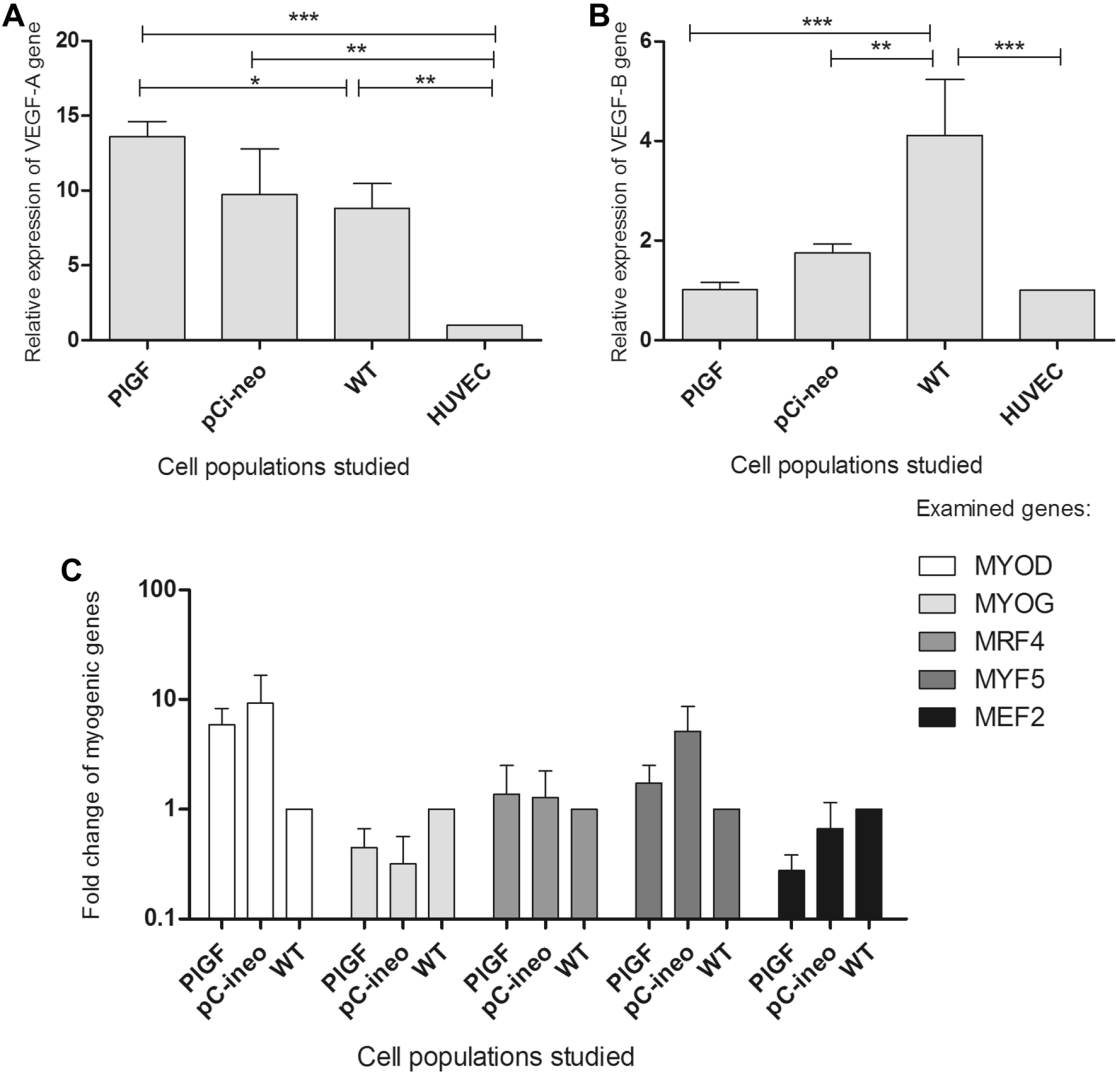
Evaluation of gene expression in myoblast populations using real-time PCR; *n* = 3 experiments. Data are presented as fold change in relative mRNA level. Gene expression was normalized to the β-actin housekeeping gene. **a** Expression of *VEGF*-*A* gene in examined myoblast populations; the observed differences between the cell populations were statistically significant in the case of HUVECs vs. native myoblasts (WT), **p < 0.01; HUVECs vs. pCi-neo-transfected cells, **p < 0.01 and HUVECs vs. *PlGF*-transfected cells, ***p < 0.001 and native cells (WT) vs. *PlGF*-transfected myoblasts *p < 0.05. **b** With respect to *VEGF*-*B* expression in cell populations, statistically significant differences were observed between the following populations: HUVECs vs. native myoblasts (WT), ***p < 0.001; native myoblasts (WT) vs. pCi-neo-transfected cells, **p < 0.01 and native myoblasts (WT) vs. *PlGF*-transfected cells, **p < 0.01. **c** Data are presented as fold change in relative mRNA level in log scale. The assessment was made using real-time PCR. There was no statistically significant difference in the expression of genes studied. The following genes were examined: *MYOD* myogenic differentiation 1, *MYOG* myogenin, *MRF4* (*MYF6*) myogenic factor 6, *MYF5* myogenic factor 5, *MEF2* myocyte enhancer factor 2

Next, we evaluated the expression of the *VEGF*-*A* and *VEGF*-*B* genes in the pCi-neo-*PlGF*-transfected myoblasts (Fig. 3). In the case of the *VEGF*-*A* isoform, we observed upregulation of this gene, particularly in the pCi-neo-*PlGF*-transfected myoblasts. We observed significantly increased *VEGF*-*A* expression levels in “native” myoblasts and pCi-neo-*PlGF*-transfected cells in comparison with that in control HUVECs (Fig. 3a). The expression of *VEGF*-*B* was significantly elevated in “native” myoblasts in comparison with that in HUVECs; however, the *PlGF*-transfected cell population appeared to have the lowest levels of *VEGF*-*B* expression among the cell populations studied, but the levels were comparable to those observed in HUVECs (Fig. 3b). However, it should be noted that there was an elevation of gene expression in cell populations transfected with empty vector.

### Myogenic Gene Expression

Quantitative assessment of myogenic gene expression did not reveal any statistically significant changes in the expression profile (Fig. 3c).

### Proliferation of Myoblast Populations

**Fig. 4:**
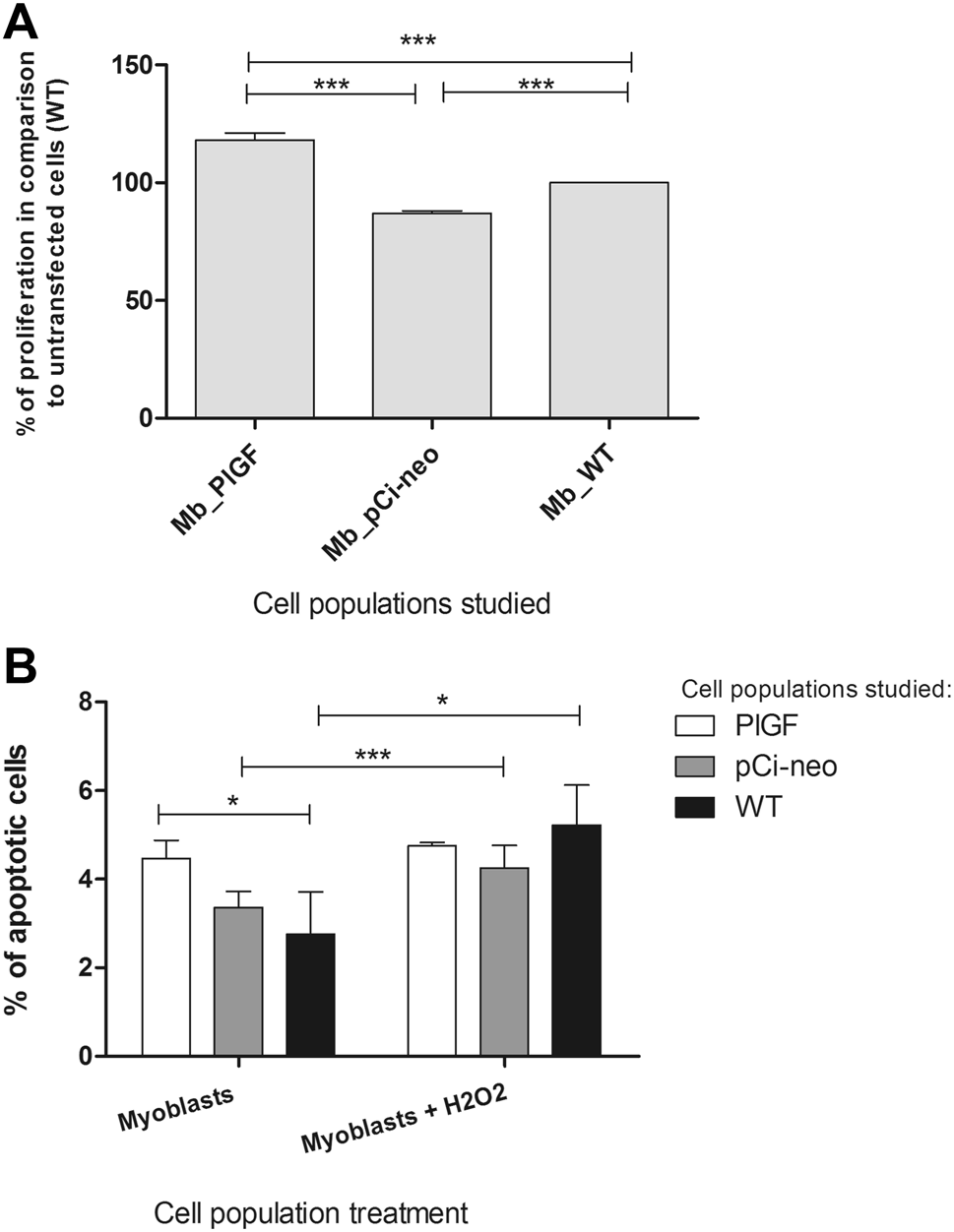
**a** Cell proliferation evaluated using MTT assay; *n* = 3 experiments. Statistically significant differences were found between the following cell populations: ***p < 0.001 *PlGF*-transfected cells vs. pCi-neo-transfected cells; ***p < 0.001 *PlGF*-transfected cells vs. native cells (WT) and ***p < 0.001 pCi-neo transfected cells vs. WT cells. **b** Percentage of apoptotic cells in the myoblast populations under standard in vitro culture conditions and subjected to 24 h of incubation with 500 μM H_2_O_2_; *n* = 3 experiments. Statistically significant differences were found between the following cell populations: *p < 0.05 *PlGF*-transfected cells vs. native cells (WT), ***p < 0.001 pCi-neo-transfected cells vs. pCi-neo-transfected cells treated with H_2_O_2_, *p < 0.05 native cells (WT) vs. native cells (WT) treated with H_2_O_2_

Myoblasts overexpressing the *PlGF* gene displayed a significantly high proliferation rate in comparison with the control and mock-transfected cells, while the human (“native”) myogenic stem cells exhibited a relatively low proliferative capacity (Fig. 4a).

### Apoptosis of Human Primary Myoblasts Subjected to Oxidative Stress Conditions In Vitro

Using flow cytometric analyses, we observed that all of the studied cell populations under both standard and oxidative stress conditions exhibited low levels of apoptosis in vitro (Fig. 4b). In the case of cells cultured under standard conditions, the apoptotic rate was slightly increased in the transfected cells. Interestingly, in response to hydrogen peroxide, the apoptotic rates of WT cells and *PlGF*-transfected cells were similar (Fig. 4b).

### Sprouting Assay

**Fig. 5:**
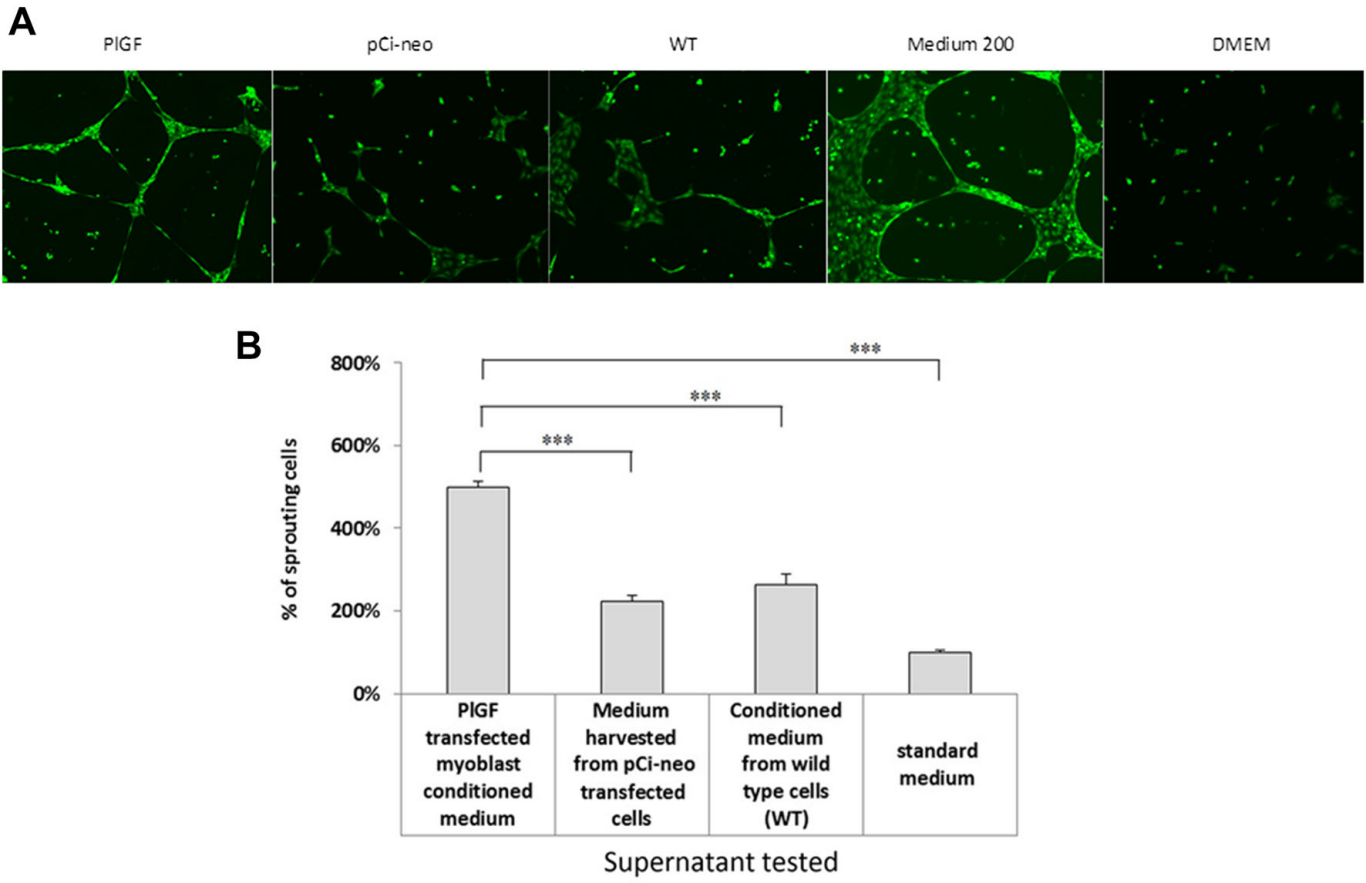
**a** Pro-angiogenic assays: images of capillaries formed upon stimulation of HUVECs with pro-angiogenic proteins in the supernatants harvested from WT cells and myoblasts transfected with *PlGF* or pCi-neo control plasmids. Medium 200 was used as a positive control and DMEM as a negative control. Sprouting assay: confirmation of pro-angiogenic properties of secreted PlGF protein. **b** Capillary formation was quantified in each experimental group and compared with capillary formation in standard medium (DMEM), set as 100%. Data are presented as the mean ± SD. Experiments were performed in triplicate for each type of medium. Statistically significant differences between supernatants from the following treatment groups were found: *PlGF*-transfected cell medium vs. pCi-neo-transfected cell medium; *PlGF*-transfected cell medium vs. WT cell medium, *PlGF*-transfected cell medium vs. standard medium (DMEM), ***p < 0.001

We confirmed the pro-angiogenic properties of PlGF secreted from transfected SkMCs by conducting a pro-angiogenic assay using HUVECs. Supernatants harvested from *PlGF*-transfected cells significantly enhanced capillary formation in HUVECs (Fig. 5a). The experiment was performed in triplicate. The total length of the capillaries was significantly higher in HUVECs treated with supernatants from the transfected cells in comparison with HUVECs treated with supernatants obtained from the in vitro cultures of either “native” cells or mock-transfected cells (Fig. 5b).

### Echocardiographic Evaluation of in Vivo Heart Function

**Fig. 6:**
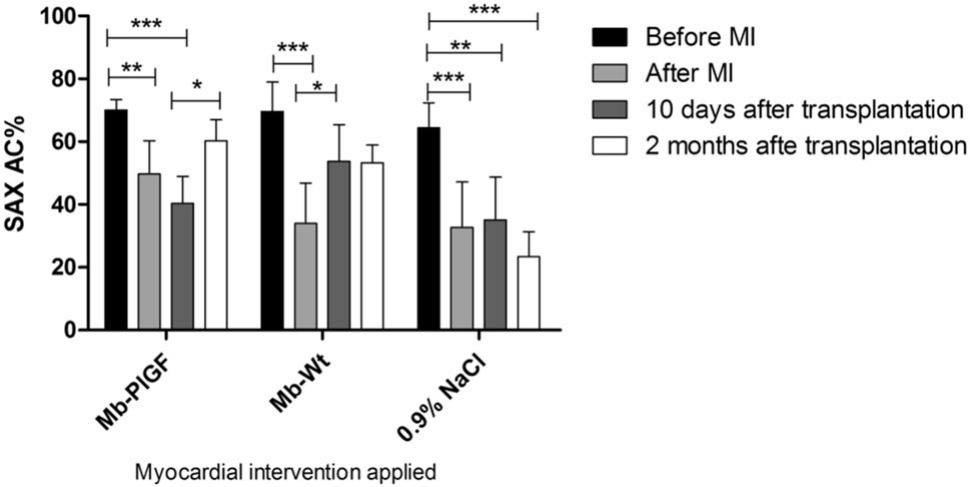
Left ventricular function: echocardiographic analysis of percentage change in short axis area (SAX AC%) at different time points—before surgical intervention, after coronary artery ligation, 10 days after cell injection/0.9% NaCl intervention and 3 months after MI induction. *Asterisks* indicate statistical significance (*p < 0.05, **p < 0.01 and ***p < 0.001). *Mb*-*PlGF* intervention with *PlGF*-transfected myoblasts, *Mb-Wt* intervention with wild-type muscle stem cells and 0.9% NaCl, injection with sodium chloride at the border zone. Experiments were performed with 12 post-infarction animals divided into three subgroups: transplanted with *PlGF*-transfected myoblasts (Mb-*PlGF*), *n* = 6; transplanted with wild-type myoblasts (Mb-Wt), *n* = 3; and injected with 0.9% NaCl, *n* = 3

Before MI induction, the SAX AC% was approximately 70%, which is characteristic of a healthy mouse heart. We observed that after coronary artery ligation the SAX AC index decreased and was followed by acute heart failure. Echocardiography showed that 2 months after *PlGF*-transfected myoblast cell transplantation, the SAX AC index was elevated, which indicated an improvement in the left ventricular function. In the case of mice treated with 0.9% NaCl, we observed a significant deterioration in cardiac function during the experimental period. During the 2 months after transplantation of WT myoblasts, we did not observe any significant changes in the SAX AC index (Fig. 6).

### Gene Expression and Histological Analysis in Collected Heart Tissues

**Fig. 7:**
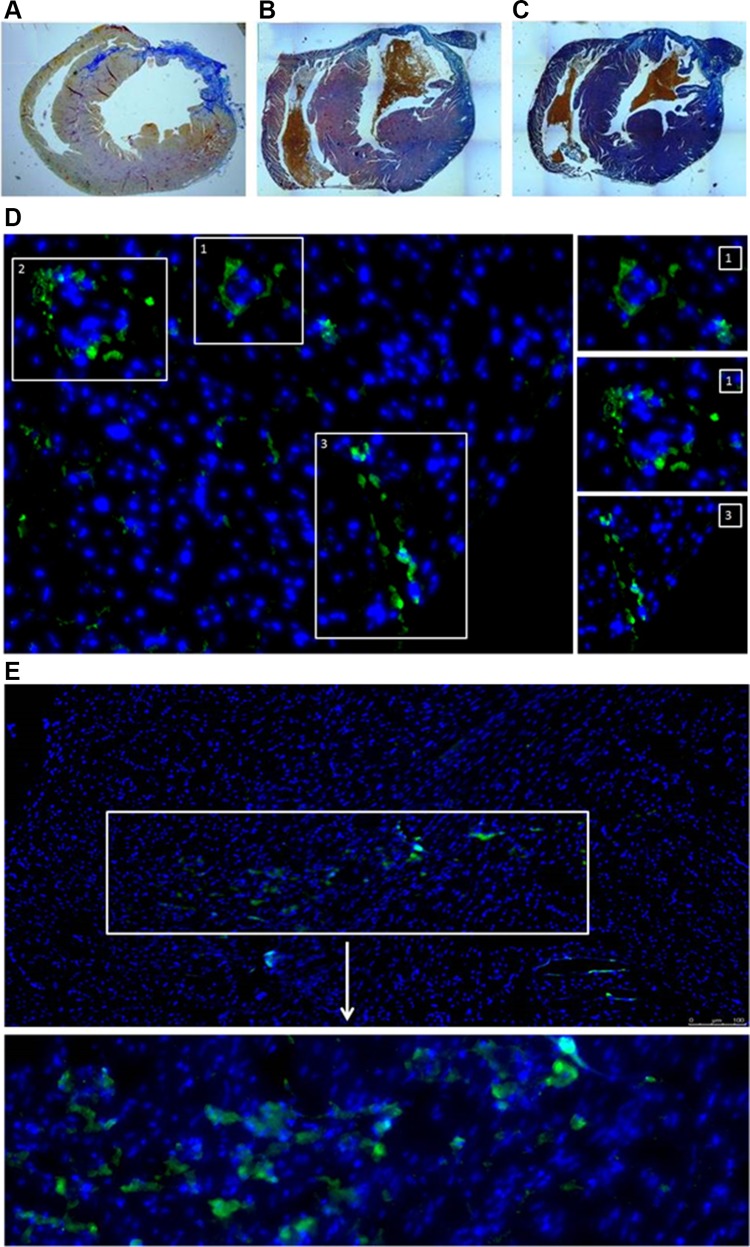
Histological analysis of heart tissue samples by trichrome staining: **a** heart after infarction, **b** heart treated with NaCl and **c** heart injected with *PlGF*-transfected myoblasts. Cell survival in isolated heart tissues; staining of human mitochondria in cells grafted into mouse heart: **d** 24 h after injection; green, Alexa Fluor 488-conjugated antimitochondrial antibody and blue, DAPI (nucleus); *panels 1, 2 and 3* show magnified images of particular sites. **e** Seven days after injection; green, Alexa Fluor 488-conjugated antimitochondrial antibody and blue, DAPI (nucleus). The analyses were performed on three post-infarcted heart samples, each representing a different treatment group: transplanted with *PlGF*-transfected myoblasts (Mb-*PlGF*), *n* = 1; transplanted with wild-type myoblasts (Mb-Wt), *n* = 1; and injected with 0.9% NaCl, *n* = 1

**Fig. 8:**
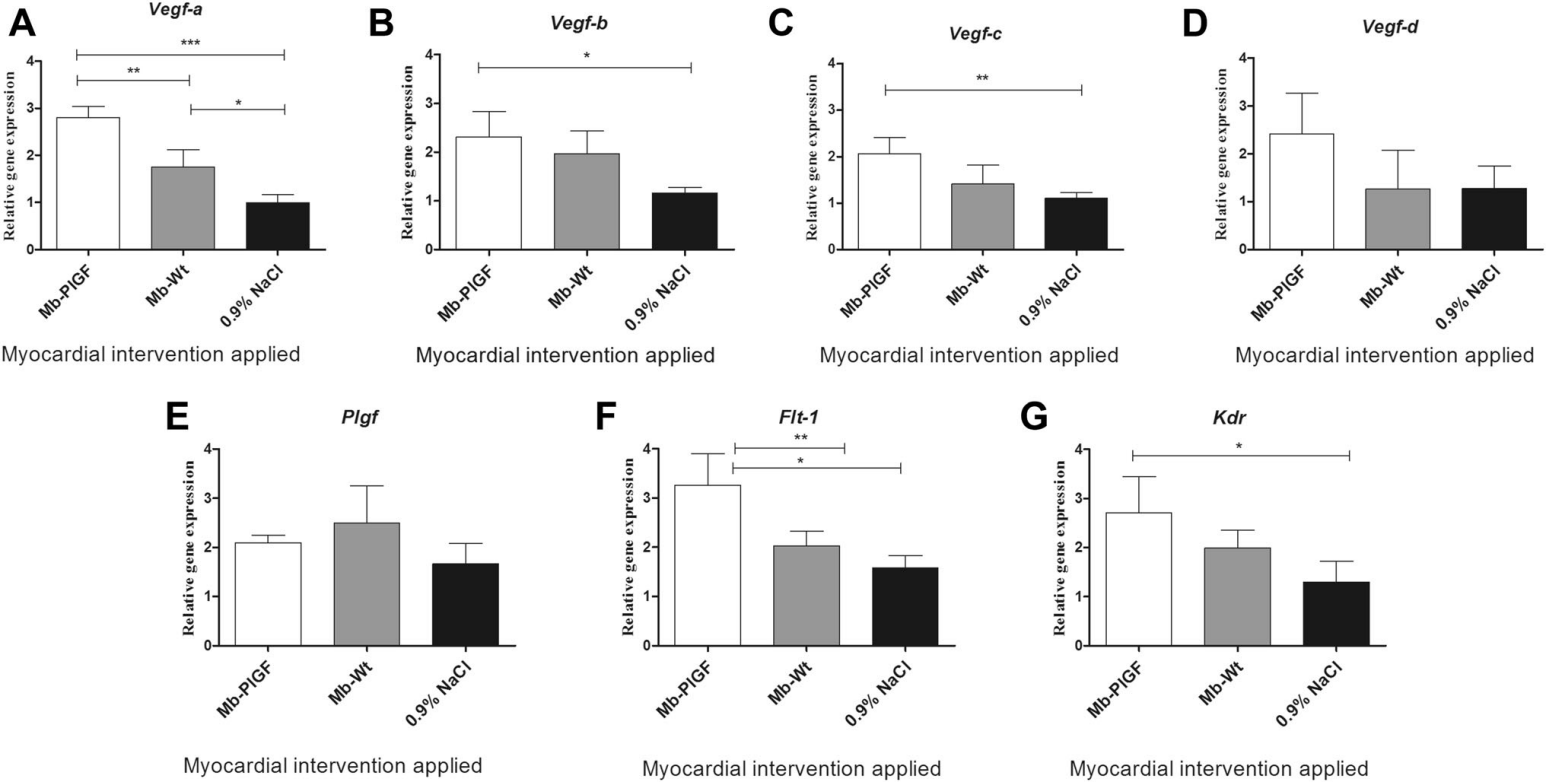
Expression of pro-angiogenic genes in the samples of post-infarcted mouse hearts treated with Mb-PlGF (*PlGF*-transfected myoblasts), Mb-Wt (wild-type myoblasts) and 0.9% NaCl. *Asterisks* indicate statistical significance (*p < 0.05, **p < 0.01 and ***p < 0.001). The following genes were evaluated: *Vegf*-*a, b, c and d*, vascular endothelial growth factor isoforms a, b, c and d, respectively; *Plgf*, placental growth factor; *Kdr*, kinase insert domain receptor and *Flt*-*1*, fms-related tyrosine kinase I receptor. Experiments were performed with 12 post-infarction animals divided into three subgroups: transplanted with *PlGF*-transfected myoblasts (Mb-*PlGF*), *n* = 6; transplanted with wild-type myoblasts (Mb-Wt), *n* = 3; and injected with 0.9% NaCl, *n* = 3

Histological analysis identified stem cells administered into the scar area (Fig. 7d, e). The scar area appeared diminished at 1 month after transplantation (Fig. 7a–c). The expression of the most prominent pro-angiogenic gene *Vegf*-*A* (Fig. 8a) was elevated in the myocardium of mice injected with genetically modified myoblasts. Interestingly, the implantation of WT myoblasts also stimulated the expression of this pro-angiogenic gene; however, the increase in expression was not as significant as that due to the injection of *PlGF*-transfected myoblasts. Additionally, the expression levels of two other *Vegf* isoforms were upregulated (*b* and *c*) (Fig. 8b, c) in the myocardium of mice that were injected with the genetically modified myoblasts. Additionally, the expression levels of the genes coding for the *Flt*-*1* and *Kdr* receptors (Fig. 8f, g) were upregulated, especially in the case of *Flt*-*1*, which is considered to be the main receptor for the examined pro-angiogenic factors. There was a slight trend towards the upregulation of the *Vegf*-*d* gene (Fig. 8d). We also examined the expression of mouse *Plgf* after therapy, but we found no connection between the transplanted human *PlGF*-transfected cells and the expression of endogenous mouse *Plgf* (Fig. 8e).

## Discussion

In the present study, we obtained *PlGF*-transfected human SkMCs. After introducing the *PlGF* gene to human myogenic stem cells, we assessed the overexpression of PlGF at both transcript and protein levels. We also examined the influence of this genetic modification on basic cellular features. Subsequently, we verified the pro-angiogenic properties of the introduced gene and the expression of other myogenic genes. Finally, after observing positive outcomes in the in vitro studies, we verified the therapeutic effect in vivo on post-infarcted hearts using a mouse model.

The efficacy of transfection of human myoblasts reached approximately 80%, which was quite satisfactory considering the fact that primary myoblasts have been shown to be relatively difficult to manipulate genetically (Green et al. 2001). In comparison with “native” cells, we observed a 50-fold increase in gene expression in the transfected cells. It has already been reported in the literature that *PlGF* transcripts can be detected in the heart, lung, thyroid and smooth muscle. Hence, there is a detectable low expression of *PlGF* in “native” human myoblasts, in which small amounts of this transcript have been found (Landgren et al. 1998). However, the observed dramatic overexpression of *PlGF* may indicate the effectiveness of electroporation as an appropriate method for genetically modifying human skeletal muscle cells.

We have also obtained high levels of PlGF protein in the supernatants of genetically modified cells in comparison with “native cells” (Fig. 2). According to the literature, we can assume that this level of expression of PlGF may be sufficient to induce effective angiogenesis. It was previously established that a dose of 100 pg of protein/ml can lead to effective capillary formation (Iwama et al. 2006). It has also been shown that the levels of PlGF in the peripheral blood of mice with acute myocardial infarction are high at 3 days after reperfusion (Ziche et al. 1997). One study showed that before reperfusion the concentration of PlGF in the artery responsible for the infarct was low and similar to that in the blood sampled from both the coronary artery (CAos) and coronary sinus (CS) (18–20 pg/ml). This concentration was equivalent to the PlGF protein levels secreted from “native” endothelial cells that form blood vessels. Interestingly, after reperfusion, the PlGF levels in the blood from the CAos and CS were significantly elevated and reached 80–90 pg/ml. This may lead to the conclusion that PlGF protein production may be enhanced to compensate for the damage exerted by myocardial infarction to initiate the formation of new capillaries (Ziche et al. 1997).

At present, we can take advantage of genetically modified stem cells in clinical trials to further pursue regenerative organ therapies. Therefore, we should concentrate not only on the implantation of genetically modified stem cells at target sites but also on the influence of additionally applied factors in healing processes such as neoangiogenesis. It is well known that PlGF can interact with VEGF protein resulting in the heterodimerization of both factors. Due to the multiplicity of PlGF and VEGF isoforms, they can give rise to many combinations, leading to effective VEGF/PlGF formation. Although this area has not been well studied so far, it has been shown in some cases that the dimerization of VEGF and PlGF can enhance angiogenesis (Cao et al. 1996; DiSalvo et al. 1995). Since this phenomenon is quite intriguing, we have examined the influence of *PlGF* overexpression on the expression of two isoforms of the pro-angiogenic factor VEGF (*VEGF*-*A* and *VEGF*-*B*). As has been previously shown, *PlGF* expression might induce the expression of other related pro-angiogenic factors such as VEGF, Ang-1 and Ang-2 (Iwasaki et al. 2011). We discovered a similar tendency only in the case of *VEGF*-*A*, whereas the expression of the *VEGF*-*B* isoform was reduced in response to *PlGF*. We can therefore assume that there is a correlation between *PlGF* and *VEGF*-*A* expression, which can potentially lead to the heterodimerization of both factors to further improve the pro-angiogenic potential of genetically modified myoblasts. Our experiments also included an empty vector as control, which could indicate the influence of the transfection process itself on myoblasts. We are aware that the process of genetic modification may cause some alterations in gene expression (this has also been shown in the case of myogenic genes, especially the *MYOD* gene). Thus, genetically modified cells should be cultured longer in vitro in order to re-establish a normal genetic profile.

It has also been documented that PlGF is mitogenic towards HUVECs and coronary venular endothelial cells and can induce the migration and growth of these endothelial cell populations (Carnaúba et al. 2011). This may be because the these cell populations possess PlGF receptors. Additionally, in our hands, the transfection of human stem cells (myoblasts) with the *PlGF* gene improved their proliferative potential (Fig. 4a). This is interesting since there is no evidence in the literature for the existence of PlGF receptors on the cell surface of myoblasts. There is, however, a possibility that the overexpression of *PlGF* might cause the enhanced expression of some genes that are involved in cell proliferation. The increase in proliferation of myoblasts may illustrate the ability of transfected human myogenic stem cells to propagate. On the other hand, it has been shown that the proliferative capacity of SkMCs can be context dependent (Perez-Ilzarbe et al. 2008). Increased but regulated proliferation of myoblasts can improve their engraftment to the scarred area, which may indirectly exert positive effect via inhibiting left ventricular remodelling. Additionally, other indirect effects include improvements in the contractile ability and ejection fraction of the heart (Gmeiner et al. 2011).

With regard to cardiac regeneration, it is very important to use cells that are highly resistant to ischaemia. Myoblasts are viable under conditions of oxygen deprivation. As illustrated in Fig. 4b, “native” myoblasts exhibit relatively low apoptosis rates under oxidative stress conditions. Moreover, the *PlGF*-transfected cells showed a similar rate of apoptosis in comparison with WT cells under hypoxic conditions. We can therefore speculate that myoblasts are good candidates for transplantation into ischaemic regions of the heart because their viability is satisfactory even under markedly reduced oxygen conditions in vitro. Additionally, we can assume that neither the electroporation process itself nor the forced overexpression of *PlGF* exerted a negative effect on the viability of the studied cells. Furthermore, we have previously learnt that *PlGF* gene transfer significantly reduces cardiomyocyte apoptosis (Iwasaki et al. 2011). Thus, PlGF can be a “survival” signal for endothelial cells, although it has also been shown to repair injured cardiomyocytes (Iwasaki et al. 2011). Presumably, myoblasts transfected with the *PlGF* gene can not only act as a graft to fill the scarred area and induce angiogenesis in the ischaemic region but also produce and secrete the PlGF factor, which can contribute to the regeneration of cardiomyocytes in close proximity of the graft (Iwasaki et al. 2011). Previous in vivo studies in rat ischaemic hearts have demonstrated the ability of myoblasts overexpressing *PlGF* to support the failing myocardium (Gmeiner et al. 2011).

Genetic modification of human myogenic stem cells may potentially disrupt myogenesis as a side effect. The principal genes involved in the maturation of myotubes can be divided into two subgroups: genes that participate in the activation of satellite cells (e.g., *MYOD* and *MYF5*) and genes that are involved in the differentiation of myoblasts into myotubes (e.g., *MYOGENIN* and *MRF4*) (Seale and Rudnicki 2000; Seale et al. 2001). We have also evaluated the transcript levels of the *MYF2* gene. Recently, it has been shown that muscle stem cells with decreased expression of *MYOD* demonstrate better therapeutic potential than “native” cells (Asakura et al. 2007). These cells undergo apoptosis at a lower rate than unmodified cells, and after engraftment into the scarred area, they tend to survive better than the other cell type (Asakura et al. 2007). In addition, myoblasts exhibiting low levels of *MYOD* can enhance the production of pro-angiogenic factors including PlGF (Nakamura et al. 2012).

To verify the pro-angiogenic properties of PlGF, we performed the sprouting assay. Placental growth factor can act through its receptor VEGF-R1. HUVECs belong to a population of endothelial cells that express the receptor for PlGF on their cell surface; thus, they can be stimulated by this ligand (Sawano et al. 1996). The binding of PlGF to its receptor induces the proliferation and migration of HUVECs. A series of experiments in our study demonstrated that conditioned media from myoblasts transfected with *PlGF* increased the number and length of capillaries formed by HUVECs (Fig. 5). Thus, we concluded that the concentration of pro-angiogenic factors in the supernatant was sufficient to stimulate angiogenesis in vitro and possibly in vivo. We can thus assume that the myoblasts secreting PlGF into the post-infarction scar will be capable of inducing capillary formation. Additionally, PlGF factor not only stimulates the formation of naked, immature vessels but also recruits pericytes and smooth muscle cells (Iwasaki et al. 2011).

The final phase of our study involved the transplantation of genetically modified myoblasts into post-infarcted mouse hearts. Administration of human recombinant PlGF has been shown to stimulate the regeneration of ischaemic heart and limbs (Autiero et al. 2003). Furthermore, it has been shown that the systemic delivery of recombinant PlGF protein after MI attenuates left ventricular remodelling and restores cardiac function by improving regional blood flow (Takeda et al. 2009). In conclusion, the benefit of PlGF on the post-ischaemic myocardium is well documented. We provided the “proof of concept” using a mouse model with human stem cells. Previous studies with ischaemic rat hearts demonstrated improved fractional shortening and ejection fraction of hearts treated with *PlGF*-transfected myoblasts. Our study showed that echocardiography of the left ventricle showed improved haemodynamic properties of the heart after therapy with both genetically modified myoblasts and WT myoblasts in contrast to treatment with 0.9% NaCl solution. A connection between functional cardiac improvement after therapy (increase in SAX AC%, Fig. 6) and expression of pro-angiogenic genes (Fig. 8) has been established. There is also an indication that pro-angiogenic factors can work synergistically (Fig. 8) and that the overexpression of any given factor may depend on the expression of other pro-angiogenic factors. In our study, after cell therapy, the most prominent pro-angiogenic genes (*Vegf*-*a, Vegf*-*b,* and *Vegf*-*c*) and their receptors (*Flt*-*1* and *Kdr*) were upregulated in the myocardium 3 months after MI induction. From the results of echocardiography, we could agree that the left ventricular function was equally improved with both WT cells and *PlGF*-transfected cells (Fig. 6). However, with regard to the gene expression profile of the scar tissue (Fig. 8), we detected higher expression of pro-angiogenic genes and their receptors in the heart tissue samples treated with *PlGF*-transfected myoblasts. Therefore, we can conclude that the myoblasts transfected with pro-angiogenic genes are more effective in rescuing the cardiac function, consistent with the results presented in Online Resource 1 in which *PlGF*-transfected myoblasts improved the functions of the left ventricle after 2 months more effectively than the “native” cells.

Interestingly, we were able to detect cells in the region of the post-infarction scar by IHC at 24 h and 7 days after transplantation. In Fig. 7d, panel 3, a needle trace covered with cells is visible, indicating that the injection was appropriately administered.

We can assume that the therapy was successful not only at the functional level but also at the molecular level in which the introduced factors could enhance the expression of pro-angiogenic genes. This synergistic effect might restore the haemodynamic properties of the heart and prevent the progression towards acute heart failure as observed in the group of mice treated with physiological saline. It is worth noting that the cellular therapy provided a prolonged benefit since the results at endpoint were assessed 3 months after MI induction, which is a significant period of time in the life of a mouse. The relatively late long-term effect upon cell intervention could be confirmed by the visible differences in the progression of cardiac remodelling with time from MI (compared to the saline-treated group from day 0 to 3 months after MI). Similarly, the effect of cellular engraftment reached statistical significance only at later stages of cardiac remodelling as shown by our previous study on human myoblasts transfected with connexin 43 (Kolanowski et al. 2014).

In conclusion, the main “proof of concept” provided by this study was the demonstration that the application of genetically modified human SkMCs with an ectopically expressed *PlGF* gene can exert a therapeutic effect on post-infarcted mouse hearts. We have assessed the influence of this pro-angiogenic gene on the basic biological features of myogenic stem cells. The overexpression of *PlGF* was increased by 50-fold, which was reflected in the secretion of biologically relevant concentrations of the functional protein. The introduction of this gene did not appear to present a negative effect on cell cycle and myogenic gene expression, but resulted in an increase in the proliferation of the stem cells. Moreover, the implemented genetic modification was therapeutically confirmed in both in vitro and in vivo systems. Therefore, we were able to show the possible therapeutic potential of genetically modified human myoblasts with the overexpression of the *PlGF* gene in a preclinical setting.

## Electronic supplementary material

Below is the link to the electronic supplementary material.

**Online Resource 1** Kinetics of cardiac haemodynamic parameters in the respective groups subjected to saline/cell interventions: (A) Before MI; (B) 28 days after MI; (C) 10 days after intervention (38 days after MI); (D) two months after intervention (three months after MI). Abbreviations: Mb-*PlGF* (*PlGF*-transfected myoblasts); Mb-Wt (wild-type myoblasts) and 0.9% NaCl (sodium chloride). Experiments were performed with 12 post-infarction animals divided into three subgroups: transplanted with *PlGF*-transfected myoblasts (Mb-*PlGF*), *n* = 6; transplanted with wild-type myoblasts (Mb-Wt), *n* = 3 and injected with 0.9% NaCl, *n* = 3 (TIFF 2747 kb)

## References

[CR1] Asakura A, Hirai H, Kablar B (2007). Increased survival of muscle stem cells lacking the MyoD gene after transplantation into regenerating skeletal muscle. Proc Natl Acad Sci USA.

[CR2] Autiero M, Luttun A, Tjwa M (2003). Placental growth factor and its receptor, vascular endothelial growth factor receptor-1: novel targets for stimulation of ischemic tissue revascularization and inhibition of angiogenic and inflammatory disorders. J Thromb Haemost.

[CR3] Becker C, Lacchini S, Muotri AR (2006). Skeletal muscle cells expressing VEGF induce capillary formation and reduce cardiac injury in rats. Int J Cardiol.

[CR4] Cao Y, Chen H, Zhou L (1996). Heterodimers of placenta growth factor/vascular endothelial growth factor. Endothelial activity, tumor cell expression, and high affinity binding to Flk-1/KDR. J Biol Chem.

[CR5] Carnaúba AT, Farias VV, Santos N (2011). Influence of gender on the vestibular evoked myogenic potential. Braz J Otorhinolaryngol.

[CR6] DiSalvo J, Bayne ML, Conn G (1995). Purification and characterization of a naturally occurring vascular endothelial growth factor-placenta growth factor heterodimer. J Biol Chem.

[CR7] Gavira JJ, Abizanda G, Caro D (2008). Skeletal myoblasts for cardiac repair in animal models. Eur Heart J Suppl.

[CR8] Gigante B, Morlino G, Gentile MT (2006). PlGF^−/−^eNos^−/−^ mice show defective angiogenesis associated with increased oxidative stress in response to tissue ischemia. FASEB J.

[CR9] Gmeiner M, Zimpfer D, Holfeld J (2011). Improvement of cardiac function in the failing rat heart after transfer of skeletal myoblasts engineered to overexpress placental growth factor. J Thorac Cardiovasc Surg.

[CR10] Green CJ, Lichtlen P, Huynh NT (2001). Placenta growth factor gene expression is induced by hypoxia in fibroblasts: a central role for metal transcription factor-1. Cancer Res.

[CR11] Haider H, Akbar SA, Ashraf M (2009). Angiomyogenesis for myocardial repair. Antioxid Redox Signal.

[CR12] Iwama H, Uemura S, Naya N (2006). Cardiac expression of placental growth factor predicts the improvement of chronic phase left ventricular function in patients with acute myocardial infarction. J Am Coll Cardiol.

[CR13] Iwasaki H, Kawamoto A, Tjwa M (2011). PlGF repairs myocardial ischemia through mechanisms of angiogenesis, cardioprotection and recruitment of myo-angiogenic competent marrow progenitors. PLoS One.

[CR14] Kawasuji M (2002). Therapeutic angiogenesis for ischemic heart disease. Ann Thorac Cardiovasc Surg.

[CR15] Kolanowski TJ, Rozwadowska N, Malcher A (2014). In vitro and in vivo characteristics of connexin 43-modified human skeletal myoblasts as candidates for prospective stem cell therapy for the failing heart. Int J Cardiol.

[CR16] Landgren E, Schiller P, Cao Y (1998). Placenta growth factor stimulates MAP kinase and mitogenicity but not phospholipase C-gamma and migration of endothelial cells expressing Flt. Oncogene.

[CR17] Maglione D, Guerriero V, Viglietto G (1991). Isolation of a human placenta cDNA coding for a protein related to the vascular permeability factor. Proc Natl Acad Sci USA.

[CR18] Menasché P (2007). Skeletal myoblasts as a therapeutic agent. Prog Cardiovasc Dis.

[CR19] Menasché P (2008). Skeletal myoblasts and cardiac repair. J Mol Cell Cardiol.

[CR20] Menasché P (2008). Skeletal myoblasts for cardiac repair: act II. J Am Coll Cardiol.

[CR21] Nakamura Y, Asakura Y, Piras BA (2012). Increased angiogenesis and improved left ventricular function after transplantation of myoblasts lacking the MyoD gene into infarcted myocardium. PLoS One.

[CR22] Perez-Ilzarbe M, Agbulut O, Pelacho B (2008). Characterization of the paracrine effects of human skeletal myoblasts transplanted in infarcted myocardium. Eur J Heart Fail.

[CR23] Rissanen TT, Ylä-Herttuala S (2007). Current status of cardiovascular gene therapy. Mol Ther.

[CR24] Roger VL, Go AS, Lloyd-Jones DM (2012). Heart disease and stroke statistics 2012-update. A report from the American Heart Association. Circulation.

[CR25] Roncal C, Buysschaert I, Chorianopoulos E (2008). Beneficial effects of prolonged systemic administration of PlGF on late outcome of post-ischaemic myocardial performance. J Pathol.

[CR26] Rozwadowska N, Fiszer D, Siminiak T (2002). Evaluation of in vitro culture of human myoblasts for tissue autotransplants to the post-infarcted heart. Pol Heart J.

[CR27] Sawano A, Takahashi T, Yamaguchi S (1996). FIt-I but not KDR/Flk-1 tyrosine kinase is a receptor for placenta growth factor, which is related to vascular endothelial growth factor. Cell Growth Differ.

[CR28] Seale P, Rudnicki MA (2000). A new look at the origin, function, and -stem-cell status of muscle satellite cells. Dev Biol.

[CR29] Seale P, Asakura A, Rudnicki MA (2001). The potential of muscle stem cells. Dev Cell.

[CR30] Takeda Y, Uemura S, Iwama H (2009). Treatment with recombinant placental growth factor (PlGF) enhances both angiogenesis and arteriogenesis and improves survival after myocardial infarction. Circ J.

[CR31] World Health Organization (2012) Cardiovascular Diseases, Statistics on chronic diseases and risk factors. http://www.who.int/topics/cardiovascular_diseases/en/index.html

[CR32] Ziche M, Maglione D, Ribatti D (1997). Placenta growth factor-1 is chemotactic, mitogenic, and angiogenic. Lab Investig.

