# Supplementary figures and images for: Biological and Pro-Angiogenic Properties of Genetically Modified Human Primary Myoblasts Overexpressing Placental Growth Factor in In Vitro and In Vivo Studies
Source: Arch Immunol Ther Exp (Warsz). 2017 Sep 26;66(2):145–59. doi: 10.1007/s00005-017-0486-2 (PMC5851700; doi:10.1007/s00005-017-0486-2)

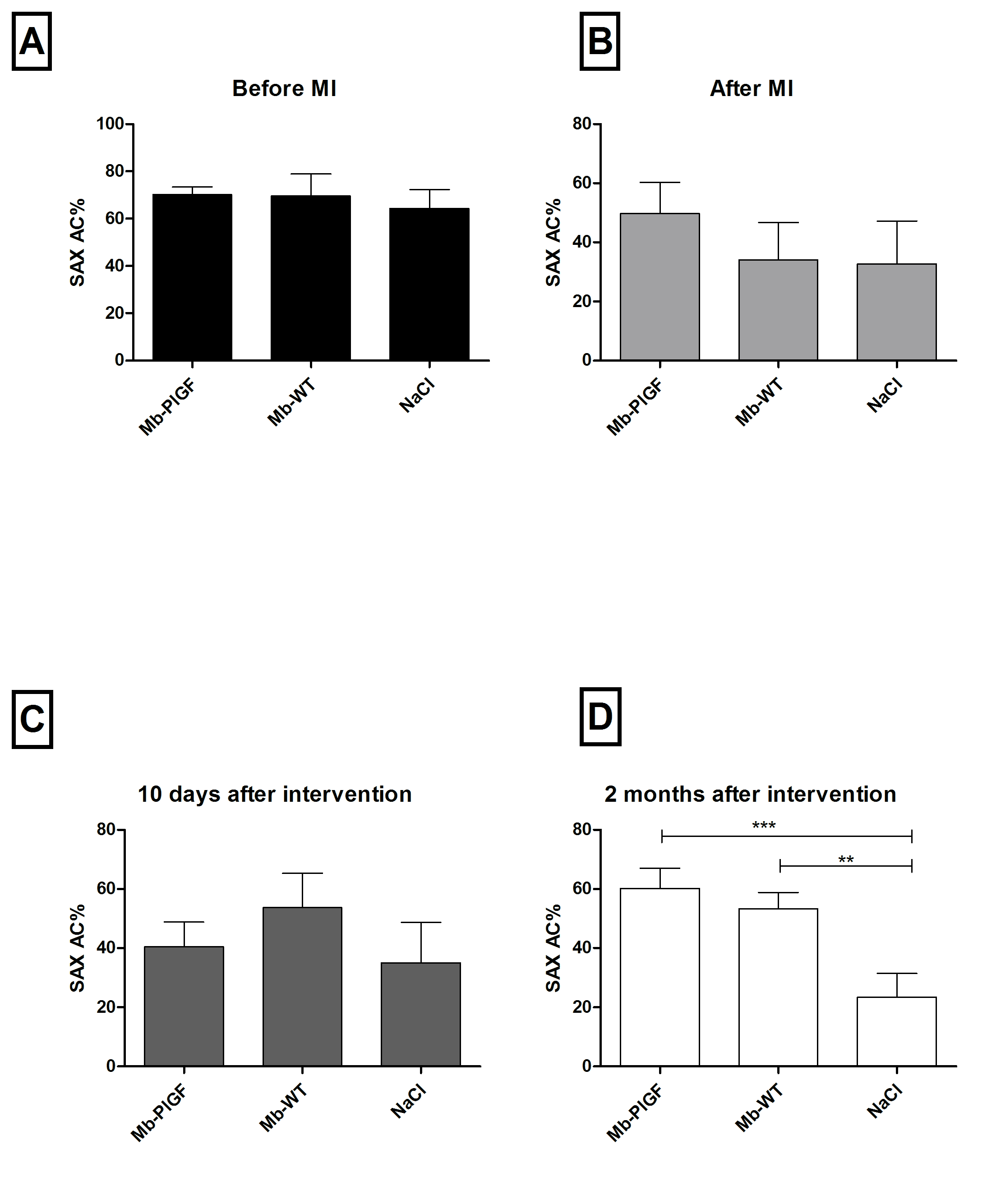

Supplement: Supplementary file 1 — Online Resource 1 Kinetics of cardiac haemodynamic parameters in the respective groups subjected to saline/cell interventions: (A) Before MI; (B) 28 days after MI; (C) 10 days after intervention (38 days after MI); (D) two months after intervention (three months after MI). Abbreviations: Mb-PlGF (PlGF-transfected myoblasts); Mb-Wt (wild-type myoblasts) and 0.9% NaCl (sodium chloride). Experiments were performed with 12 post-infarction animals divided into three subgroups: transplanted with PlGF-transfected myoblasts (Mb-PlGF), n = 6; transplanted with wild-type myoblasts (Mb-Wt), n = 3 and injected with 0.9% NaCl, n = 3 (TIFF 2747 kb) [file 5_2017_486_MOESM1_ESM.tif]
